# Assessment of the Efficacy of Home Remedial Methods to Improve Drinking Water Quality in Two Major Aquifer Systems in Jaffna Peninsula, Sri Lanka

**DOI:** 10.1155/2017/9478589

**Published:** 2017-10-18

**Authors:** W. M. Dimuthu Nilmini Wijeyaratne, Suvendran Subanky

**Affiliations:** Department of Zoology and Environmental Management, Faculty of Science, University of Kelaniya, Kelaniya, Sri Lanka

## Abstract

Chunnakam and Vadamaradchi are two major aquifer systems in Jaffna Peninsula, Sri Lanka. This study was performed to compare water quality in the domestic wells in these aquifers and to assess the efficacy of household water treatments for treating contaminated water. Replicate well water samples were collected from each aquifer and pH, dissolved oxygen (DO), conductivity, total dissolved solids (TDS), salinity, temperature, total solids (TS), total hardness (TH), chemical oxygen demand (COD), oil and grease (OG), nitrate N (N), and total phosphate (TP) were measured. The sampled water from the domestic wells was filtered through commercial mineral filter and* Moringa oleifera* leaf powder and boiled at 100°C for 10 minutes and the TH, OG, N, and TP were measured. Both OG and N in Chunnakam were significantly higher and the DO were significantly lower than those of Vadamaradchi. TH, N, and OG of some wells exceeded the drinking water quality standards established by Sri Lanka Standards Institution.* Moringa oleifera* leaf powder filtration reduced N significantly and filtering through commercial mineral filter reduced OG, TH, and N significantly. Boiling at 100°C could remove TH significantly but may cause significant increase in N which might result in health impacts.

## 1. Introduction

Jaffna Peninsula which is located in the northernmost region of Sri Lanka contains four main aquifer systems, namely, Chunnakam (Valikamam area), Thenmaradchi, Vadamaradchi, and Kayts [1]. The geology of this area is mainly composed of miocene limestone that is considered to be appropriate geological material for the development of aquifers [2]. There are no perennial rivers in this part of the country due to flatness of the land and lack of rainfall. Due to flat topography of this area the construction of reservoirs is impossible. Therefore, all the domestic water and industrial and agricultural requirements are substantially dependent on the groundwater sources. The recharge of these groundwater sources occurs during very short rainy season [3]. There are over 100,000 dug wells in the Peninsula, of which approximately 20% are farm wells and the remaining ones are used to meet requirements for domestic and drinking purposes in urban and rural areas [4].

The groundwater resources of the peninsula remain almost constant while the demand for water continues to increase with increasing population and development activities. Furthermore, due to uneven distribution of rainfall in time and space, water resources are declining. The management of groundwater resources has not been accompanied by systematic studies to evaluate potential of the existing groundwater resources [5]. Continuation of overextraction exceeding the safe yield may subsequently lead to progressive decline in groundwater table [6]. 

The groundwater aquifers in Jaffna Peninsula are also prone to groundwater quality problems, as the resource is limited and its quality has deteriorated over time with nonsustainable extraction. Salt water intrusion due to overextraction, sewage contamination due to improper soakage pits, hardness of water, contamination with nitrate due to overuse of fertilizer, and contamination due to crude oil have become major concerns affecting the quality of available groundwater [7–9]. Crude oil contamination of groundwater in Jaffna Peninsula has been a concern since 2008. When the water is contaminated with crude oil it becomes undrinkable due to the oily odour and the presence of potential toxic hydrocarbon products and heavy metals in crude oil can cause serious health effects as well [9, 10]. Despite the presence of high nitrate, oil, and grease levels, the households in these areas solely rely on groundwater for domestic and drinking purposes. Thus it is essential to provide cheaper, feasible, and efficient household water treatments to purify the water collected from the domestic wells before using them for domestic and drinking needs.

The remedial actions to make the contaminated water in a potable condition are extensively investigated around the world. Various natural and synthetic remediation methods have been studied to improve the quality of the contaminated water in different parts of the world. The use of natural materials is increasingly popular among many research groups as these methods provide more cost-effective, environmentally friendly methods of water purification. Papaya seeds with clay,* Moringa oleifera* seeds, and* Moringa oleifera* leaf extract have been studied for their removal efficiency of contaminants [11–14]. The adsorption of copper from aqueous solution using papaya seed as a low-cost adsorbent was investigated and it was found that papaya seed has high potential to be employed as an effective adsorbent in removing copper ions and would be useful for the design of wastewater treatment plants for heavy metal removal [15].

The* Moringa oleifera* leaf extract has shown successful results in domestic wastewater treatment to reduce hardness and total dissolved solids to improve the quality of water. Also it has been found that leaves of* Moringa oleifera* have the capacity to reduce sulphate and nitrate concentration in the water [16]. However, use of these simple yet effective treatment methods is not widely researched in Sri Lanka.

This study mainly focussed on assessment of water quality in Chunnakam and Vadamaradchi aquifers in Jaffna Peninsula. Vadamaradchi aquifer is considered as the most uncontaminated aquifer in Jaffna Peninsula and the Chunnakam aquifer is the largest and main lime stone aquifer of Jaffna Peninsula and it is considered to be highly contaminated with nitrate due to excessive usage of fertilizers. Further, people complain about water becoming unsuitable for domestic and agricultural purposes due to contamination with crude oil. People in the Chunnakam area are supplied with potable water from noncontaminated aquifers at Vadamaradchi area as a temporary solution. But there are no proper studies carried out to compare the water quality parameters in Chunnakam aquifer with the water from uncontaminated aquifers in Jaffna Peninsula. In addition to that there are no remedial actions suggested for purification of contaminated water in order to improve the water quality.

Therefore, this study was performed to compare the concentration of oil and grease, nitrate N, total solids, total hardness, and total phosphate and other basic water quality parameters in the water collected from domestic wells located at Chunnakam and Vadamaradchi aquifers and to identify the efficacy of small scale home remedial measures for treating contaminated water.

## 2. Methodology

### 2.1. Study Area

This sampling sites were located in Vadamaradchi and Chunnakam Divisional Secretariat Divisions in Jaffna Peninsula. The Vadamaradchi area is fed by the Vadamaradchi aquifer and the Chunnakam area is fed by the Chunnakam aquifer.

**Figure 1:**
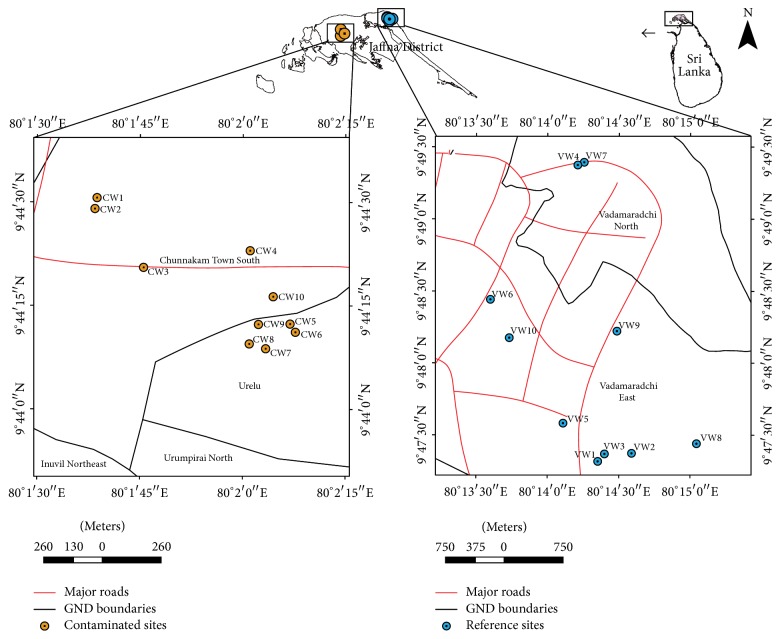
Map of the study area showing the sampled wells. (CW: wells in the Chunnakam area; VW: wells in the Vadamaradchi area).

From each aquifer, 10 domestic wells were randomly selected and five replicate water samples were collected from each well and preserved in accordance with APHA, 1998 [17]. The location of the sampled domestic wells is given in Figure 1.

### 2.2. Water Quality Analysis

The temperature, salinity, pH, electrical conductivity, dissolved oxygen concentration, and total dissolved solids of each water sample were measured in situ using precalibrated multiparameter water quality checker (HACH model: H940). The water samples were preserved in accordance with APHA, 1998 [17], and were transported to the laboratory of the Department of Zoology and Environmental Management, University of Kelaniya, Sri Lanka. In the laboratory chemical oxygen demand (COD), nitrate concentration, total phosphorus concentration, total hardness, total solids, and oil and grease concentration were measured following the methodologies described in APHA, 1998. In order to assess the spatial variation of water quality parameters, sampling at each well (Figure 1) was done at monthly intervals from May to August 2016.

### 2.3. Water Treatment Assays

The sampled water from the domestic wells (Figure 1) was subjected to filtering through commercial mineral filter, filtering through* Moringa oleifera* leaf powder (1 g/L of raw water in a glass filtration column), and boiling at 100°C for 10 minutes and the total hardness, oil and grease concentration, nitrate N concentration, and total phosphorous concentration were measured in these water samples, following the methodologies described in APHA, 1998 [17].

*Moringa oleifera* leaf powder was prepared as described by Saduzaman et al., 2013. The* Moringa oleifera* leaves were harvested from the* Moringa* tree and rinsed with distilled water. Then it was shade-dried and shade-dried leaves were ground into powder form using grinder (Bright Elegant-240V6A). The resulting powder was stored in a desiccator at a cool dry place until used in the filtration experiment.

### 2.4. Statistical Analysis

After confirming the normality using Anderson darling test, the data were analysed using Student's* t*-test to determine the significance of the spatial variation of water quality parameters among the domestic wells of Vadamaradchi and Chunnakam areas. Paired* t*-test was used to analyse the significance of differences in water quality parameters before and after the treatments. MINITAB 14 statistical software package was used in the statistical analysis.

### 2.5. Comparison with Drinking Water Quality Standards

The measured physicochemical parameters in domestic wells before and after treatment were compared with the drinking water quality standards established by Sri Lanka Standards Institution (SLSI).

## 3. Results and Discussion

### 3.1. Water Quality Analysis

Spatial variation of mean ± standard deviation (SD) and the minimum and maximum values of water quality parameters sampled from the domestic wells in Vadamaradchi area and Chunnakam areas and the drinking water standards established by the Sri Lanka Standards Institution [18, 19] and the percentage of sampled wells exceeding SLSI drinking water standards are given in Table 1.

The pH, electrical conductivity, total dissolved solids, salinity, temperature, total solids, total hardness, chemical oxygen demand, and total phosphorous concentrations in the domestic wells of Chunnakam area were not significantly different from those of the Vadamaradchi area (Table 1). The oil and grease concentration and the nitrate concentration in the water collected from the domestic wells in the Chunnakam area were significantly higher and the dissolved oxygen concentration was significantly lower compared to that of the Vadamaradchi area (Table 1). The source of oil and grease contamination is unknown and the increased nitrate levels in the groundwater in the Chunnakam area can account for the extensive usage of nitrogen fertilizer in this area during the agricultural activities. Further, the degradation of high concentrations of nitrate and oil and grease by aerobic bacteria may have caused a significant reduction of dissolved oxygen concentration in the Chunnakam area compared to that of the Vadamaradchi area.

The pH, total dissolved solids, electrical conductivity, and total phosphorous values recorded during the study are within the SLSI standards for safe drinking water. The upper limit in the range of the total hardness values in the domestic well water recorded during the study in both Chunnakam and Vadamaradchi areas exceeds the standard total hardness levels for safe drinking water established by SLSI. Four categories of water hardness can be identified based on the CaCO_3_ hardness [20]: CaCO_3_ hardness below 60 mg/L: soft; 60–120 mg/L: moderately hard; 120–180 mg/L: hard; and more than 180 mg/ L: very hard water. The mean CaCO_3_ hardness values recorded from the domestic wells in Chunnakam and Vadamaradchi areas are 225 mg/L and 254.4 mg/L, respectively (Table 1). These results indicate that drinking water in both these areas can be categorized as very hard water. However, the range of CaCO_3_ hardness values recorded from the domestic wells in Chunnakam and Vadamaradchi areas (Table 1) indicates that the level of hardness in the drinking water in this area ranges from moderate to very hard. The total hardness in well water in Jaffna Peninsula is relatively high due to the presence of calcium, magnesium, chloride, and sulphate ions [6]. Calcium is the major cause of high hardness levels in groundwater in Jaffna Peninsula [6]. Calcium and magnesium are primarily found in groundwater due to the dissolution of limestone and the substantial contribution from the weathering of rocks depending on the interaction of other factors, such as pH and alkalinity [2]. Although the residents of Jaffna Peninsula consume water with high levels of dissolved solids and hardness over the decades, water exceeding the drinking water standards cannot be recommended for consumption without treatment as hard water may cause formation of bladder stones with the higher phosphate concentration. Out of sampled wells, 20% of the wells showed total hardness values above the drinking water standards (Table 1).

The groundwater of Jaffna Peninsula has the highest nitrate content in Sri Lanka. A study in Chunnakam aquifer showed that the nitrate N concentration ranged from 0 to 35 mg/L [8]. During the present study nitrate N ranges from 3.11 to 40.01 mg/L in Chunnakam. The most probable cause for the high concentrations of nitrate in wells could be the excess fertilizers leached to the shallow groundwater. The calcic soil favours the leaching of nitrate N as it has high permeability [21]. The addition of abundant nitrogenous waste and synthetic and animal fertilizers is identified as the major cause for nitrate contamination. The proximity of toilets and poor design of soakage pits and wells also may contribute to the high nitrogen levels in the wells [8].

In the present study, 30% of the sampled wells were above the limit for standard level of nitrate established by SLSI [18, 19] (Table 1) and all of them were located in Chunnakam. These wells were located close to the densely cultivated lands. Water with high levels of nitrogen, exceeding the drinking water standards, cannot be recommended for consumption without treatment as it has potential to cause severe health effects as the high number of stomach cancers and malignant tumours were related to elevated nitrate in Jaffna [22, 23].

The oil and grease concentration was significantly higher in Chunnakam area than the Vadamaradchi area (Table 1). Seven wells showed oil and grease concentrations higher than the SLSI standard for drinking water and all these wells were located in the Chunnakam area. The oil and grease concentration in the domestic wells sampled from Vadamaradchi were less than the SLSI standard for drinking water. These results indicate the possibility of having a nonpoint or point source of oil and grease in Chunnakam. However, the exact cause of elevated oil and grease concentrations in the Chunnakam area is unknown.

### 3.2. Water Treatment Assays

The variation of mean ± SD of total hardness, nitrate N concentration, total phosphate concentrations, and oil and grease concentration of water after each treatment are given in Table 2. Total hardness of the water samples was significantly decreased after filtration through the commercial mineral filter and boiling of water at 100°C for 10 minutes, while it was significantly increased after filtration through* Moringa oleifera* leaf powder (Table 2). However, significant reduction of nitrate N concentration could be observed after filtering through* Moringa oleifera* leaf powder. Significant reduction of nitrate N concentration was also observed after filtering through the commercial mineral filter. But boiling water at 100°C for 10 minutes resulted in significant increase in nitrate N concentration. Significant reduction in oil and grease concentration could be observed after filtering through the commercial mineral filter and boiling water at 100°C for 10 minutes. However, filtration through* Moringa oleifera* leaf powder did not reduce the oil and grease concentration (Table 2).

The* Moringa oleifera* leaf powder filter causes reduction of nitrate significantly as the positively charged proteins in* Moringa oleifera *leaves provide surface for adsorption of negatively charged nitrate [16]. Therefore, there is a possibility of using the coagulant capability of* Moringa oleifera* leaf powder in small scale to reduce nitrate ion concentration as the* Moringa oleifera* tree is widely grown in tropical countries including Sri Lanka. The significant increase of nitrate N concentration after boiling of water at 100°C for 10 minutes may be due to concentration of ions after evaporation of water at the boiling point.

Filtering through the commercial mineral filter decreased the oil and grease concentration significantly and it may have resulted from adsorption of oil and grease onto mineral surfaces of the filter [16]. However, filtering through the* Moringa oleifera *leaf powder did not show a significant difference in oil and grease concentration (Table 2). There was a significant decrease in oil and grease after boiling at 100°C for 10 minutes. This may be due to volatilization of volatile compound in crude oil or degradation of oil and grease.

## 4. Conclusion

The water sampled from the domestic wells located in Chunnakam in Jaffna Peninsula, Sri Lanka, cannot be used for drinking and other domestic uses unless they are subjected to proper treatment to make sure the water quality is within the accepted standard levels. The commercial mineral filter is suitable for reducing total phosphorous, oil and grease, total hardness, and nitrate N concentration in the water. Boiling at 100°C for 10 minutes could be used to remove significant amount of total hardness in drinking water but that may cause significant increase in nitrate concentration which might result in severe health impacts. Therefore, boiling is not preferred for the water collected from wells in Chunnakam which has comparatively elevated nitrate N concentration.* Moringa oleifera *leaf powder can be used as a home remedial method to treat water with elevated nitrate N concentrations and it would provide a cheaper and efficient option as for nitrate removal. However, as the filtration by* Moringa oleifera *leaf powder significantly increases the hardness of water, it is recommended to combine this treatment with hardness reduction treatments in order to improve the usability of the treatment.

## Figures and Tables

**Table 1:** Mean ± SD of water quality parameters of the domestic wells in Vadamaradchi and Chunnakam areas during the study period, drinking water standards established by the Sri Lanka Standards Institution (SLSI 614, 2013 [18, 19]), and the percentage of sampled wells exceeding SLSI drinking water standards. In Vadamaradchi and Chunnakam columns, different superscripts (*∗* and *∗∗*) at each row (for each water quality parameter) indicate significant differences from each other at 95% level of significance (*p* < 0.05, Student's *t*-test, *n* = 10). The range of water quality parameters of the domestic wells in Vadamaradchi and Chunnakam areas is given within parentheses.

Parameter	Vadamaradchi	Chunnakam	SLSI drinking water standards	Percentage of wells exceeding the drinking water standards (%)
pH	7.93 ± 2.1^*∗*^ (7.53–8.33)	7.38 ± 0.1^*∗*^ (7.03–7.57)	6.5–8.5	0
DO (mg/L)	7.27 ± 0.8^*∗*^ (6.89–8.60)	5.94 ± 1.1^*∗∗*^ (5.0 to 8.6)	Not mentioned	—
EC (*µ*S/cm)	824.2 ± 217.5^*∗*^ (168.8–905.0)	692.1 ± 80.6^*∗*^ (503.0–950.3)	Not mentioned	—
TDS (mg/L)	405.6 ± 109.7^*∗*^ (80.1–758.2)	338.4 ± 30.8^*∗*^ (247.3–467.2)	500	0
Salinity (‰)	0.37 ± 0.09^*∗*^ (0.08–0.58)	0.34 ± 0.03^*∗*^ (0.24 to 0.46)	Not mentioned	—
Temperature (°C)	29.82 ± 0.2^*∗*^ (29.53–30.03)	30.05 ± 0.4^*∗*^ (29.72–30.1)	Not mentioned	—
Total solids (mg/L)	568.0 ± 169.1^*∗*^ (95.6–957.8)	533.1 ± 63.2^*∗*^ (445.3 to 654.0)	Not mentioned	—
Total hardness (mg/L, CaCO_3_)	254.4 ± 46.1^*∗*^ (67.5–332.3)	225.0 ± 31.6^*∗*^ (192–252.5)	250	20
COD (mg/L)	7.55 ± 0.51^*∗*^ (4.65–9.97)	6.27 ± 0.51^*∗*^ (4.09–7.89)	10	0
Oil & grease (mg/L)	0.023 ± 0.00^*∗*^ (0.011–0.033)	0.248 ± 0.030^*∗∗*^ (0.113–0.373)	0.2	35
Nitrate N (mg/L)	1.69 ± 0.46^*∗*^ (0.021–7.87)	16.28 ± 2.46^*∗∗*^ (3.11–40.1)	11.2	30
Total phosphate (mg/L)	0.090 ± 0.005^*∗*^ (0.07–0.113)	0.078 ± 0.005^*∗*^ (0.05–0.113)	2.0	0

**Table 2:** Mean values ± SD of physicochemical parameters of raw water and treated water at each treatment. The mean values indicated by different superscript letters at each row are significantly different from each other (paired *t*-test, *p* < 0.05, *n* = 20).

	Raw water	Commercial mineral filter	*Moringa oleifera* leaf powder filter	Boiling at 100°C for 10 minutes
Total hardness (mg/L, CaCO_3_)	249.4 ± 7.6^a^	221.8 ± 11.2^b^	296.1 ± 14.3^c^	228.9 ± 7.7^b^
Oil & grease (mg/L)	0.135 ± 0.046^a^	0.035 ± 0.005^b^	0.149 ± 0.008^a^	0.045 ± 0.007^b^
Nitrate N (mg/L)	8.98 ± 1.10^a^	6.15 ± 1.81^b^	7.72 ± 2.19^b^	10.05 ± 0.64^c^
Total phosphate (mg/L)	0.083 ± 0.006^a^	0.051 ± 0.006^b^	0.100 ± 0.007^a^	0.072 ± 0.004^a^
